# What are the effects of supporting early parenting by newborn behavioral observations (NBO)? A cluster randomised trial

**DOI:** 10.1186/s40359-020-00467-5

**Published:** 2020-10-16

**Authors:** Ingeborg Hedegaard Kristensen, Svend Juul, Hanne Kronborg

**Affiliations:** 1 Nursing and Health Care, Department of Public Health, Aarhus University, Bartholins Allé 2, 8000 Aarhus C, Denmark; 2 Epidemiology, Department of Public Health, Aarhus University, Bartholins Allé 2, 8000 Aarhus C, Denmark

**Keywords:** Parenting intervention, Newborn behavioral observations, NBO, Early intervention, Universal intervention, Health visitor, Parent-infant relationship, Community setting, CARE-index

## Abstract

**Background:**

Professional support to enhance the early parent-infant relationship in the first months after birth is recommended, but little is known about the effect of universal interventions. The objective was to investigate the effect of health visitors’ use of the Newborn Behavioral Observations system in new families.

**Methods:**

A cluster-randomised study was conducted in four Danish municipalities. Health visitors’ geographical districts constituted the units for randomisation (*n* = 17). In the intervention group, 1332 families received NBO from 3 weeks after birth; in the comparison group, 1234 received usual care. Self-administered questionnaires were collected at baseline one to two weeks after birth, and at follow-up three and nine months postpartum. The outcomes were change over time measured by The Karitane Parenting Confidence Scale (KPCS), The Major Depression Inventory (MDI), The Ages and Stages Questionnaire: social-emotional (ASQ:SE) and The Mother and Baby Interaction Scale (MABIC). Data were analysed with mixed-effects linear regression using the intention-to-treat approach.

**Results:**

At baseline, no significant differences between the two groups were seen regarding maternal and infant factors. At follow-up three and nine months after birth, the change in maternal confidence and mood, infant’s socio-emotional behaviour, and early parent-infant relationship moved in a slightly more positive direction in the intervention group than in the comparison group, though not statistically significant. The only significant effect was that the intervention mothers reported higher level of knowledge about infant’s communication skills, response to cues, and how to sooth and establish a relation with the infant, compared to the comparison group.

**Conclusions:**

We found no effect of the NBO system delivered in a universal context to all families in a community setting. The only significant difference between groups was a higher maternal degree of knowledge regarding early parenting in the intervention group.

**Trial registration:**

ClinicalTrials.gov ID: NCT03070652. Registrated February 22, 2017.

## Background

The early parent-infant relationship affects the later physical and psychosocial health and cognitive development of the child [1–5]. Thus, the World Health Organization (WHO) recommends that early parental support is delivered universally by health professionals in the form of home visits if possible [6]. In a new family, parental sensitivity and responsiveness to the infant are likely to affect the quality of the early parent-infant relationship [7–9]. Parents’ experience of insecurity and problems such as the infant persistently crying or having interrupted sleep [10–13] may influence this early relationship and affect parental mental health after birth [14–16]. More than half of first-time mothers have reported a need for support in the early postpartum period [17, 18], and in recent research, one in five first-time mothers showed signs of low parenting confidence or symptoms of depression [18, 19].

Knowledge is scarce on the effect of universal programmes addressing the early parent-infant relationship offered to a general population of new parents. A recent review of universal home visiting interventions delivered by health visitors targeting new families, found small but positive effects in three of six outcomes; maternal life course, child cognitive and language skills, and parental behaviour and skills [20]. Parenting interventions by addressing parents´ sensitivity and responsiveness in the interaction with their infants have been shown to have a positive impact on the parents’ psychosocial functioning, parent-infant interaction and infant development when targeting selected at risk families characteristised as being either teen parents, having symptoms of depression or highly reactive infants [21–23].

Newborn Behavioural Observations (NBO) is an 18-item neuro-behavioural intervention including observations to enhance the parent–infant relationship [20]. The NBO has until now been tested in clinical settings in a general population of new families in Norway [24] and in selected at risk families in the UK, China and the US [25–27]. These previous studies have found that NBO helped mothers learn about infants’ early cues among new families in general [24], and it increased the quality of care related to parent-infant interaction among at risk families [25, 27] and reduced symptoms in depressive mothers [26]. A Cochrane review which did not include the latest study [24] found relatively low-quality evidence in these studies of the effectiveness of the NBO [28]. A qualitative study among mothers of preterm infants concluded that NBO may favour the mother’s understanding of the behaviour of the newborn and her participation in care [29]. The NBO, which is widely used in the US, the UK and a number of European countries, has not yet been tested in a community setting in a general population of new parents.

### Aim and hypothesis

The aim of this study was to evaluate the effects on maternal, infant and relationship outcomes of the implementation of the NBO system by health visitors in a general population of families in a community setting. We hypothesised that early maternal support, facilitated by the standardised NBO system, would increase maternal confidence and mood, and that provision of early maternal support would improve infant socio-emotional behaviour as well as the early mother-infant relationship during the first months after birth compared with provision of standard care to families from health visitors.

## Methods

### Design and setting

A cluster-randomised study with two parallel arms examined the effect of the NBO system in a community setting. The study was conducted in four Danish municipalities, representing 17 geographical districts, 111 health visitors and a total population of 396,000 inhabitants. In Denmark, health visitors provide early parenting support to approximately 97% of all new families, mostly by home visits [30]. Danish health visitors are registered nurses with a 18-month further education in promoting maternal, child and family health [30]. The early intervention is not standardised, but the Danish Health Authority recommends that health visitors focus on the families’ health, child development, and the establishment of an early healthy parent-infant relationship [30].

In January 2016, the health visitors in the participating municipalities were informed about the study; in June 2016, the 17 geographical health visitor districts were randomised to either the intervention or the comparison group.

### Recruitment and participants

Recruitment of new families was initiated 1 January 2017 by health visitors and continued until 31 January 2018. At the first home visit after birth, health visitors in both groups invited all families with a newborn to participate in the study. The oral invitation was followed by written information about the study, and oral and written informed consent was subsequently obtained from the participating mothers. All mothers who were visited by a health visitor 1–2 weeks after birth were eligible. We had no exclusion criteria except mothers who were undergoing treatment elsewhere and not living at home or who were unable to manage their own legal affairs and therefore not visited by a health visitor.

### Intervention

The NBO developed by Nugent et al. from the Brazelton Institute [31], is a standardised system with the purpose to enhance the parents’ understanding of the newborn’s cues and thereby respond sensitively to their newborn’s expressions and cues. The intervention focuses on observation of the infant combined with active involvement of the parents in a shared dialogue based on 18 neuro-behavioural items including both observation and elicited maneuvers to identify newborn behaviours and interpreting these in the context of parent-infant interaction. The items include infant habituation to stimuli, infant motor development, observation of the infant’s state regulation (consciousness from deep sleeping to crying) and response to face, voice, stress and activity using the NBO system [31]. Administration of the NBO is flexible; if the infant sleeps at the beginning of the session, then the NBO begins with the discussion of habituation issues. If, however, the infant is crying, then the session begins with observation of infants state regulation and soothability [31]. The NBO observation takes around 12 to 25 min [25, 27].

In the intervention group, families received NBO intervention practiced by the health visitors during the home visit when the infant was 3 weeks old and in any subsequent home visit until the age of 3 months [31]. In the comparison group, families received practice as usual as examination of the newborn infant already was part of health visitors’ practice during home visits after birth [30].

Health visiting practice in both groups complied with recommendations of the Danish Health Authority.

### NBO course, training, certification and supervision

Two Danish NBO trainers certified by the Brazelton Institute, UK, delivered the NBO education and supervision to all health visitors employed in the intervention districts. The aim of the NBO course, training and certification was to educate health visitors to deliver the NBO, to involve and share the observations of the 18 neuro-behavioural items of the infant with the parents and thereby enhance the parents’ sensitive and responsive interaction with their infant [31]. In September 2016, health visitors in the intervention group joined a two-day course on the background and content of the NBO system including oral presentations and video films. An interactive form with discussions, using dolls and role-plays was used to identify the infant’s strengths and vulnerabilities within the 18 neuro-behavioural items. These items were discussed in detail and a family with a newborn infant was invited for a live session of the NBO intervention. The live session was conducted by the NBO trainer followed by the observers of participating health visitors completing the NBO recording form for the live session. In connection with the course, participants received the Danish work manual, the NBO manual, “Understanding Newborn Behaviour and Early Relationship” by Nugent et al. (2007) and NBO tools. The course was followed by a two-month training phase where health visitors in their daily work practised the NBO in at least five new families. After having completed the course and practical training, 56 health visitors achieved the NBO certification in December 2016.

Supervision was given to health visitors in the intervention group during the implementation period for a total of 2 days to ensure that the delivery of NBO did not deviate from the standardised NBO system [32]. Each supervision session contained a brief presentation of the 18 neuro-behavioural items using an interactive teaching method to share observations of infant small cues, to discuss the infant’s ability to self-regulate and to support the parent-infant relationship. A project group consisting of the health visitors’ team leaders from the four municipalities, an NBO trainer and researchers met regularly during the intervention period to bridge the gap between health visitors and researchers.

### Data collection

Data were collected from 15 January 2017 until 30 November 2018. The outcomes were measured within three main domains: Maternal characteristics concerning confidence and mood development over time, Infant characteristics and social and emotional development over time and Mother-infant interaction. Mothers (*n* = 2566) received self-administered questionnaires at baseline one to two weeks after birth, and at follow-up three and nine months postpartum. The questionnaires consisted mainly of previously validated scales and questions used in earlier studies [33] and were collected via a web-based system with a personal login (TrialPartner). Reminders were sent twice, first by text message and afterwards by e-mail. Eight times, lottery prizes representing a value of 2000 DKK were drawn from the pool of participants completing the questionnaire.

### Outcomes

*The Karitane Parenting Confidence Scale (KPCS)* by Črnčec et al. (2008) [34] consists of a 15-item questionnaire measuring parenting confidence in mothers to infants. The KPCS has been validated in an Australian setting showing a good sensitivity (86%) and specificity (88%) [34]. Each item is rated on a scale from 0 to 3 and values are summed up to a KPCS score (0–45); high scores are favourable.

*The Ages and Stages Questionnaire: social-emotional (ASQ:SE)* by Squires et al. (1997) [35] consists of questionnaires with 16, 23 and 27 items measuring the infant’s socio-emotional behaviour by self-regulation, compliance, adaptive functioning, autonomy, affect, social communication and parent-child interaction. The ASQ:SE has been validated in several countries and shown a moderate to good sensitivity (71–85%) and an excellent specificity of (90–98%) [36–38]. Each item is rated on a three-point scale, and the ASQ:SE score is calculated ranging from 0 to 15; low scores are favourable.

*The Major Depression Inventory (MDI)* by Olsen et al. (2003) [39] consists of a 10-item questionnaire measuring parental symptoms of depression [40]. Each statement is rated on a three-point scale, and values are summed up to the MDI score (0–30); low scores are favourable [39].

*The Mother and Baby Interaction Scale (MABIC)* by Hackney (1996) [41] is a questionnaire with 10 items measuring the mother-infant relationship. Each statement is rated on a four-point scale and values are summed up to the MABIC score (0–40); low scores are favourable [42].

*Single item questions* assessing maternal knowledge, consisting of 5 items measuring mother’s knowledge concerning caring for the infant with regard to the infant’s communication skills, how to respond to cues, and how to establish a relation, sooth the infant and to regulate infant’s sleep were collected at one time point at the first follow-up. Each variable was rated on a five-point scale with low scores being favourable.

### Background and process variables

Background variables concerning maternal age, marital status, educational level, employment status, and the infant with regard to place of birth, gestational age, sex, and infant health are presented in Table 1. Process variables concerning mother’s perceived support from health professionals measured at first follow-up appear in Table 3.

**Table 1:** Maternal, infant and relationship factors at baseline in the intervention and comparison group

	Intervention group *n* = 1332		Comparison group *n* = 1234		
	n	Mean (S.D.) (%)	n	Mean (S.D.) (%)	*P-*value
**Maternal factors**					
Age (years)	1166	30.41 (4.64)	995	30.13 (4.84)	0.54^1^
Short education	1164	(42%)	983	(37%)	0.15^2^
Single living	1156	(4%)	983	(4%)	0.93^2^
Parity first-time	1150	(45%)	976	(48%)	0.32^2^
Parenting Confidence Scale (KPCS)^a^ < 40	1088	(24%)	926	(21%)	0.11^2^
Major Depression Inventory (MDI)^b^ > 14	1084	(9%)	914	(8%)	0.47^2^
**Infant factors**					
Preterm (less than 37 gestational weeks)	1150	(3%)	976	(4%)	0.33^2^
Age & Stages Questionnaire: (ASQ:SE)^b^ > 24	1109	(56%)	918	(53%)	0.10^2^
Average Age & Stages Questionnaire: (ASQ:SE)^b^	1095	1.67 (0.91)	932	1.63 (0.90)	0.23^1^
**Relationship factors**					
Mother and Baby Interaction Scale (MABISC)^b^	1085	8.48 (4.29)	909	8.35 (4.28)	0.46^1^

Note ^a^high score favourable ^b^low score favourable. Mixed-effects regression^1^ or logistic mixed-effects regression^2^ analysis adjusted for clustering.

### Randomisation

The randomisation was carried out in June 2016, before the health visitors’ enrolment in the NBO course. The 17 health visitor districts were allocated to the intervention or the comparison group. Prior investigation of the districts revealed that all districts represented a variation in social status among inhabitants. Because of the small number of clusters, a restricted randomisation procedure was used to achieve balance between the two study arms [43]. The purpose of the restricted randomisation was to ensure that both treatments appeared in each municipality and that the intervention and comparison group had approximately the same expected number of births. In each municipality, the districts were divided into two groups and allocated randomly to one of the two groups. An independent data manager handled the randomisation procedure.

### Statistical analysis

We performed a power analysis using the following decisions and assumptions. The design would be clustered with nine and eight districts, respectively. We wanted to demonstrate a significant difference between intervention and comparison families, provided an actual difference in KPCS score change of at least one point. We assumed the standard deviation of the KPCS score change would be four points, and we assumed the intraclass coefficient (the correlation between families within clusters) would be 0.01. To obtain a power of 90%, the average cluster size should be 66 [40].

In the analysis of data, we used the intention-to-treat analysis as our main approach, analysing data according to the families’ residence in an intervention or a comparison district. First, a baseline comparison of characteristics between the two groups was tested with mixed-effects regression for continuous variables and with logistic mixed-effects regression for categorical variables. Due to the clustered design, analyses were adjusted for two levels of clustering 1) health visitors and 2) districts. Next, the differences in change from baseline to first and second follow-up of the outcome variables, KPCS, MDI, ASQ:SE and MABIC were analysed with mixed-effects regression analysis including adjustment for clustering, parity, educational level, single living, and preterm birth. In the analysis of variables from the video recordings, we used the subsample from the intervention and comparison groups analysed by mixed-effects regressions adjusted for clustering. If more than two outcome variables were missing, the observation was excluded.

*P*-values below 0.05 were considered significant. All data were entered in TrialPartner database at Aarhus University, and the Stata software version 15.0 (StataCorp LLP, College Station, TX, USA) was used for all statistical analysis.

## Results

### Study profile

**Fig. 1:**
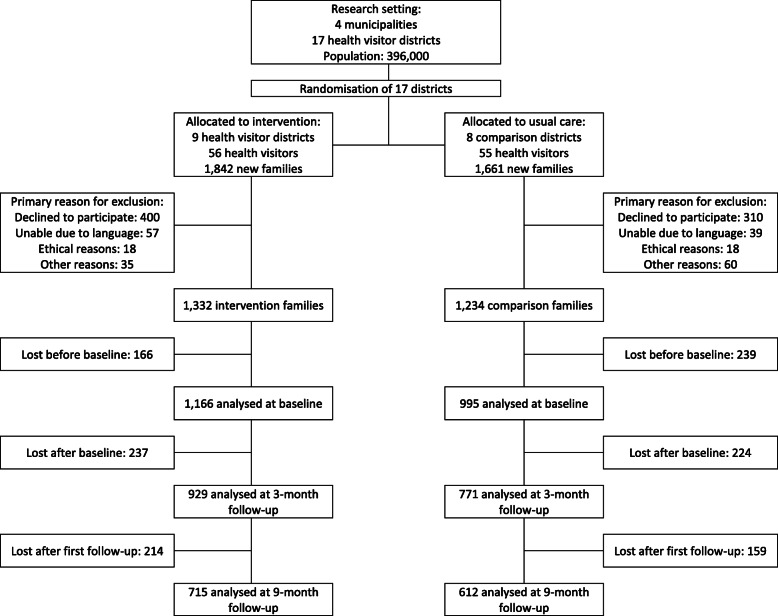
Flow diagram

The 17 health visitor districts were randomised into nine intervention districts with 56 health visitors and 1842 new families and eight comparison districts with 55 health visitors and 1661 new families. A flow profile of the study population is shown in Fig. 1. Data from 1132 intervention and 1234 comparison families were analysed at baseline. Data from 929 and 771 families were analysed at first follow–up, and 715 and 612 at second follow-up.

### Baseline characteristics

Table 1 provides baseline descriptive statistics for mothers allocated to the intervention and comparison groups. In the study population, 21–24% of the mothers had a low confidence score, 8–9% had symptoms of depression, and 3–4% of the infants were born preterm. There were no significant differences between the two groups at baseline 2 weeks after birth with regard to maternal, infant, and relationship factors.

### Difference between groups in change from baseline to first and second follow-up

Table 2 shows the difference in change of the variables KPCS, MDI, MABISC and ASQ:SE from baseline 1–2 weeks after birth to the first follow-up 3 months after birth and to the second follow-up 9 months after birth. The scores tended to improve both in the intervention and the comparison group; however, differences between changes in the two groups were small and insignificant.

**Table 2:** Change from baseline to first and second follow-up of outcomes in the intervention and comparison group

	Mean change from baseline to follow-up					
	n	Intervention	Comparison	Difference (95% CI)		*P-*value
**Follow-up 3 months postpartum**						
Maternal Confidence KPCS^a^ (0–45)	1133	0.86	0.91	0.10	(−0.48, 0.69)	0.73
Major Depression Inventory MDI^b^ (0–30)	1519	−0.68	− 0.44	− 0.39	(−1.45, 0.70	0.46
Mother and Baby Interaction Scale MABISC^b^ (0–40)	1125	−1.04	−0.79	− 0.27	(− 0.62, 0.09)	0.14
Ages & Stages Questionnaire ASQ:SE^b^ (0–15)	1178	0.29	0.29	0.00	(−0.09, 0.09)	0.98
**Follow-up 9 months postpartum**						
Maternal Confidence KPCS^a^ (0–45)	1133	1.04	0.99	0.07	(−0.68, 0.83)	0.84
Major Depression Inventory MDI^b^ (0–30)	1125	−0.17	0.07	−0.68	(−2.09, 0.74)	0.35
Mother and Baby Interaction Scale MABISC^b^ (0–40)	1125	−1.80	−1.87	−0.15	(−0.67, 0.31)	0.58
Ages & Stages Questionnaire ASQ:SE^b^ (0–15)	1178	−0.44	−0.36	− 0.08	(− 0.20, 0.04)	0.21

Note; ^a^high score favourable, ^b^low score favourable. Pp: postpartum. (95% CI): 95% confidence interval. The difference is estimated by mixed-effects regression adjusted for clustering, parity, education, single living, and preterm birth.

In a supplementary per protocol analysis using the actual allocation to a health visitor with or without NBO certification, no significant results were seen in change from baseline to first and second follow-up for the outcome variables KPCS, MDI, MABISC and ASQ:SE. Moreover, an additional mixed-effects logistic regression analysis of dichotomized outcome of the variables KPCS, MDI, MABISC and ASQ:SE showed no significant differences neither in intention-to treat nor per protocol approach analysis.

Table 3 provides information on mothers’ report about their knowledge of the infant and the perceived support from health professionals at first follow-up. The mothers from the intervention group reported a significantly higher knowledge score than the comparison group concerning the infant’s communication skills, response to infant cues, as well as how to sooth and establish a relation with the infant.

**Table 3:** Mothers’ reported knowledge and perceived support from health professionals in the intervention and comparison group

First follow-up three months after birth	Intervention group *N* = 929		Comparison group *N* = 771		
	n	Mean (S.D.) (%)	n	Mean (S.D.) (%)	*P-*value
**Mothers’ reported knowledge**					
Infant’s communication skills^a^(0–4)	859	2.01 (0.91)	700	2.18 (0.99)	**0.015**^**1**^
How to respond to infant cues^a^(0–4)	859	2.07 (0.92)	700	2.22 (0.98)	**0.036**^**1**^
How to sooth the infant^a^(0–4)	859	2.26 (1.05)	700	2.43 (1.08)	**0.007**^**1**^
How to establish a relation with the infant^a^(0–4)	859	2.11 (1.01)	700	2.26 (1.04)	**0.043**^**1**^
How to regulate infant’s sleep ^a^(0–4)	859	2.61 (1.02)	700	2.59 (1.06)	0.68^1^
**Support from health professionals**					
Infant examined three weeks postpartum	854	(94%)	701	(95%)	0.71^2^
Observations shared with health visitor	810	(94%)	663	(93%)	0.84^2^
Number of home visits by health visitor	848	3.92 (1.34)	695	3.75 (1.20)	0.11^1^
Days before first home visit by health visitor	826	6.03 (4.75)	680	5.96 (5.78)	0.91^1^
Help from health visitors by phone	857	(11%)	699	(10%)	0.92^2^
Help from general practitioner	857	(37%)	699	(35%)	0.31^2^

Note: Bold values indicate a significance level at 5%. ^a^low score favourable. Mixed-effects regression^1^ and mixed-effects logistic regression^2^analysis adjusted for clustering

### Perceived support from health professionals

Almost all mothers (94–95%), had experienced health visitors examine their infant 3 weeks after birth, and 93–94% of the mothers had shared their observations with the health visitors. Both groups of families had received an average of four home visits within 3 months after birth and no significant differences were seen in mothers´ perception of the help received from health professionals in the two groups as presented in Table 3.

### The attrition problem

Among the families invited, 72.3% in the intervention districts and 74.3% in the comparison districts agreed to participate at baseline; the difference was not significant, *P* = 0.18. To study attrition after baseline, we used a completed KPCS questionnaire as an indicator for participation. Among those who responded to the KPCS questionnaire at baseline, 76.4% also responded at first follow-up and 56.4% at second follow-up, with little difference between groups; *P* ≈ 0.50.

Table 1 shows characteristics of participants at baseline. In an additional Table 1, we show selected baseline information among participants and dropouts in the intervention and comparison group, respectively. The main contrast concerns the level of education with a higher dropout rate among women with short education; this was especially prominent in the intervention group, and at the 3-month follow-up. The contrast in educational level between participants and dropouts was significantly larger in the intervention group than in the comparison group (*P* = 0.03).

### Post-hoc power analysis

The observed intraclass correlation (ICC) for KPCS at second follow-up was 0.001 (95% CI: 0.000–0.007); the average cluster size at second follow-up was 66, and the standard deviation for the KPCS change from baseline to second follow up was 3.33 points. In a post-hoc power analysis, we used the upper ICC limit and the actual cluster size and standard deviation. In this analysis, the power to detect a difference in KPCS score change of at least one point was 0.98.

## Discussion

This study evaluated the effects of implementing the NBO system provided by health visitors as a universal intervention to all new families in a community setting. Effects on infant, mother, and interaction outcomes were assessed. Although mothers in the intervention group reported to have more knowledge than the comparison group at first follow-up about infants’ communication skills, response to infant cues, as well as how to sooth and establish a relation with the infant, we found no significant differences between groups concerning maternal confidence and symptoms of depression, infant socio-emotional behaviour, nor in early mother-infant relationship at both follow-ups.

The cluster randomised design ensured a comparison group for testing the NBO system in a universal population of parents reflecting the Danish background population. The randomisation procedure reflected a trade-off between the optimal individual randomisation procedure and what was possible in a community setting. Although some mothers dropped out from baseline to first and second follow-up, the study had a sufficient power to detect even a small effect of the intervention. The outcome scales KPCS, MDI, ASQ:SE, and MABIC were developed for use mainly in selected vulnerable families, and they have been thoroughly validated [34, 37, 40, 42]. As the outcomes of interest, we calculated the score changes from baseline to follow-up.

It is a weakness of the study that the dropout rate before the follow-up examinations was rather high, thus generating missing information on key outcomes for many families. There was an overweight of mothers with short education among dropouts compared to participants, especially in the intervention group. There were, however, only small and insignificant differences in KPCS score change between mothers with short and longer education, both in the intervention and the comparison group. Thus, we conclude that the attrition has only introduced a minor bias of unknown direction in the comparison between the groups.

We found no evidence of any effect on maternal, infant and relationship factors of the NBO system when delivered to a general population of mothers who had given birth recently, but mothers who had received NBO had more knowledge about how to communicate and respond to infant cues. The findings in this study that NBO certified health visitors may improve maternal knowledge and understanding of the newborn after attending an NBO intervention are consistent with the results from a recent Norwegian study testing the NBO system delivered by midwives and health visitors to a general population in a clinical setting [24]. The Norwegian study found that the participating mothers had learned significantly more about their infants’ cues, but no significant differences were seen between the groups for mother-infant relationship or maternal mood, respectively [24]. The findings from our present study and the Norwegian study [24] are also consistent with a previous randomised study of NBO delivered to selected families with at risk newborns finding no effect on the mother-infant relationship [25]. Furthermore, a qualitative study among mothers of preterm infants concluded that NBO may favour maternal understanding of the behaviour of the newborn and her participation in care [29]. In a previous paper [32], we investigated what the participating health visitors in the intervention group in the present study had learned after attending the NBO education programme and after having delivered the intervention. We found superior knowledge, especially about infant’s self-regulation, among health visitors in the intervention group [32]. Another cross-sectional study found that health professionals had a better understanding of the infant’s communication skills and of how to sooth the infant after participating in the NBO education programme [44]. Our present study reflected that this added knowledge may be passed on from health visitors to mothers, but we could not demonstrate any measurable effect on maternal, infant and relationship factors [8, 10].

The findings of reduced risk of maternal depressive symptoms in another study testing the NBO system [26] may be explained by the difference in the study population with a varied general population in the present study compared to a selected study population with special characteristics and therefore with special needs for health care [26]. Additionally, the delivered intervention varies in time and intensity in the studies. In the present study, a relatively short intervention was delivered 3 weeks after birth, administered at least once (one to three sessions) in the first 3 months of the infant’s life. In prior studies evaluating the effect of NBO in selected families, NBO was administered in two to seven sessions with the first session initiated a few hours after birth [26, 45]. The intervention in the present study was delivered 3 weeks after birth because the first weeks were used to collect baseline data. It has been documented that the effects of health visitor delivered home visits are associated with the number and duration of visits and with the health visitor’s education [46, 47]. In this study, the comparison group received usual care by well-educated Danish health visitors. The families in the intervention and comparison groups received the same number of home visits. The same number of days passed from birth to the first home visit, and access to health visitors and general practitioners was the same. We cannot rule out that the high level of access to healthcare could be the reason for the lack of effect of the NBO intervention in this study, showing no significant difference between the health benefits received by families in the intervention and comparison group.

## Conclusion

This is the first study of the effect of the NBO system administered as home visits in a community setting as a universal intervention for new families. The mothers in the intervention group reported to have better knowledge about the infant’s communication skills, response to cues, and about how to sooth and establish a relation with the infant than the comparison group; however, this was not reflected in the self-administered questionnaire on maternal confidence, mood, the infant socio-emotional behaviour, or the early mother-infant relationship.

## Supplementary information

**Additional file 1.**

## Data Availability

The raw data for this study are stored and handled according to the Danish Data Protection Agency’s provisions on a secure electronic drive at Aarhus University where the researchers are employed. As the data include confidential patient data, these data are not available for public access.

## Abbreviations

ASQ:SE: The Ages and Stages Questionnaire: Social-emotional
CI: Confidence interval
KPCS: The Karitane Parenting Confidence Scale
MABISC: The Mother and Baby Interaction Scale
MDI: The Major Depression Inventory
NBO: Newborn Behavioral Observations

## References

[1] Jee SH, Conn AM, Szilagyi PG, Blumkin A, Baldwin CD, Szilagyi MA (2010). Identification of social-emotional problems among young children in foster care. J Child Psychol Psychiatry.

[2] Feldman R (2007). Parent-infant synchrony and the construction of shared timing; physiological precursors, developmental outcomes, and risk conditions. J Child Psychol Psychiatry.

[3] Sroufe LA (2005). Attachment and development: a prospective, longitudinal study from birth to adulthood. Attach Hum Dev.

[4] Weinfield NS, Sroufe LA, Egeland B (2000). Attachment from infancy to early adulthood in a high-risk sample: continuity, discontinuity, and their correlates. Child Dev.

[5] Mackes NK, Golm D, Sarkar S, Kumsta R, Rutter M, Fairchild G (2020). Early childhood deprivation is associated with alterations in adult brain structure despite subsequent environmental enrichment. Proc Natl Acad Sci U S A.

[6] WHO. Child and adolescent mental health policies and plans: WHO; 2012. https://www.who.int/mental_health/maternal-child/child_adolescent/en/.

[7] Parfitt Y, Pike A, Ayers S (2013). The impact of parents’ mental health on parent–baby interaction: a prospective study. Infant Behav Dev.

[8] Kenny M, Conroy S, Pariante CM, Seneviratne G, Pawlby S (2013). Mother-infant interaction in mother and baby unit patients: before and after treatment. J Psychiatr Res.

[9] Raby KL, Roisman GI, Fraley RC, Simpson JA (2015). The enduring predictive significance of early maternal sensitivity: social and academic competence through age 32 years. Child Dev.

[10] Oldbury S, Adams K (2015). The impact of infant crying on the parent-infant relationship. Community Pract.

[11] Palmstierna P, Sepa A, Ludvigsson J (2008). Parent perceptions of child sleep: a study of 10,000 Swedish children. Acta Paediatr.

[12] Papousek M, von Hofacker N (1998). Persistent crying in early infancy: a non-trivial condition of risk for the developing mother-infant relationship. Child Care Health Dev.

[13] Lindberg B, Ohrling K (2008). Experiences of having a prematurely born infant from the perspective of mothers in northern Sweden. Int J Circumpolar Health.

[14] Field T (2010). Postpartum depression effects on early interactions, parenting, and safety practices: a review. Infant Behavior Dev JID - 7806016.

[15] Murray L, Cooper P, Fearon P (2014). Parenting difficulties and postnatal depression: implications for primary healthcare assessment and intervention. Community Pract.

[16] Lilja G, Edhborg M, Nissen E (2011). Depressive mood in women at childbirth predicts their mood and relationship with infant and partner during the first year postpartum. Scand J Caring Sci.

[17] Jones TL, Prinz RJ (2005). Potential roles of parental self-efficacy in parent and child adjustment: a review. Clin Psychol Rev.

[18] Kristensen IH, Simonsen M, Trillingsgaard T, Pontoppidan M, Kronborg H (2018). First-time mothers’ confidence mood and stress in the first months postpartum. A cohort study. Sex Reprod Healthc.

[19] Kronborg H, Væth M, Kristensen I (2012). The effect of early postpartum home visits by health visitors: a natural experiment. Public Health Nurs.

[20] Filene JH, Kaminski JW, Valle LA, Cachat P (2013). Components associated with home visiting program outcomes: a meta-analysis. Pediatrics.

[21] Barlow J, Smailagic N, Bennett C, Huband N, Jones H, Coren E. Individual and group based parenting programmes for improving psychosocial outcomes for teenage parents and their children. Cochrane Database Syst Rev. 2011;2011(3):CD002964. 10.1002/14651858.CD002964.pub2. 10.1002/14651858.CD002964.pub2 PMC4164375 21412881

[22] Velderman MK, Bakermans-Kranenburg M, Juffer F, van IJzendoorn H. (2006). M. Effects of attachment-based interventions on maternal sensitivity and infant attachment: differential susceptibility of highly reactive infants. J Fam Psychol.

[23] van Doesum KTM, Riksen-Walraven JM, Hosman CMH, Hoefnagels C (2008). A randomized controlled trial of a home-visiting intervention aimed at preventing relationship problems in depressed mothers and their infants. Child Dev.

[24] Høifødt RS, Nordahl D, Landsem IP, Csifcsák G, Bohne A, Pfuhl G, et al. Newborn behavioral observation, maternal stress, depressive symptoms and the mother-infant relationship: results from the northern babies longitudinal study (NorBaby). BMC Psychiatry. 2020;20(1):300. 10.1186/s12888-020-02669-y. 10.1186/s12888-020-02669-y PMC7294655 32539729

[25] McManus BM, Nugent JK (2014). A neurobehavioral intervention incorporated into a state early intervention program is associated with higher perceived quality of care among parents of high-risk newborns. J Behav Health Serv Res.

[26] Nugent JK, Bartlett JD, Valim C (2014). Effects of an infant-focused relationship-based hospital and home visiting intervention on reducing symptoms of postpartum maternal depression a pilot study. Infants Young Child.

[27] Nugent JK, Bartlett JD, Von Ende A, Valim C (2015). The effects of the newborn behavioral observations (NBO) system on sensitivity in mother–infant interactions. Infants Young Child.

[28] Barlow J, Herath NI, Bartram Torrance C, Bennett C, Wei Y. The neonatal behavioral assessment scale (NBAS) and newborn behavioral observations (NBO) system for supporting caregivers and improving outcomes in caregivers and their infants. Cochrane Database Syst Rev. 2018;3. 10.1002/14651858.CD011754.pub2 PMC6494209 29537066

[29] Dittz ES, Alves CRL, Duarte ED, Magalhães LDC (2017). Contribution of the newborn behavioral observations (NBO) for the maternal care of preterm neonates. J Hum Growth Dev.

[30] Poulsen A, Brot C. Vejledning om forebyggende sundhedsydelser til børn og unge [guidelines on Prevenive health schemes for children and Adolescrnts]. Kbh: Sundhedsstyrelsen. 2011;3:9–146.

[31] Nugent JK. Understanding newborn behavior & early relationships: the newborn behavioral observations (NBO) system handbook: Paul H. Brookes Pub. 2007;1:0-280.

[32] Kristensen IH, Vinter M, Nickell IK, Kronborg H. Health visitors’ competences before and after implementing the newborn behavioral observations (NBO) system in a community setting: a cluster randomised study. Public Health Nurs. 2019;36. 10.1111/phn.12658 31475391

[33] Kristensen IHIH, Simonsen M, Trillingsgaard T, Kronborg H (2017). Video feedback promotes early relations between infants and vulnerable first-time mothers. A quasi-experimental study. BMC Pregnancy Childbirth.

[34] Crncec R, Barnett B, Matthey S (2008). Development of an instrument to assess perceived self-efficacy in the parents of infants. Res Nurs Health.

[35] Squires, J., Bricker, D., Twombly, E. (2003). The ASQ:SE user’s guide. Baltimore: Paul H. Brookes Publishing Co. Vol 3. page 0-192.

[36] Squires J, Bricker D, Twombly E. The ASQ:SE user’s guide. Baltimore: Paul H. Brookes Publishing Co.; 2003. Vol 3. page 0-192.

[37] Singh A, Squires J, Yeh C, Heo K, Bian H (2016). Validity and reliability of the developmental assessment screening scale. J Fam Med Prim Care.

[38] Anunciação L, Squires J, Clifford J, Landeira-Fernandez J (2019). Confirmatory analysis and normative tables for the Brazilian ages and stages questionnaires : social–emotional. Child Care Health Dev.

[39] Olsen LR, Jensen DV, Noerholm V, Martiny K, Bech P (2003). The internal and external validity of the major depression inventory in measuring severity of depressive states. Psychol Med.

[40] Campbell MK, Grimshaw JM, Elbourne DR (2004). Intracluster correlation coefficients in cluster randomized trials: empirical insights into how should they be reported. BMC Med Res Methodol.

[41] Hackney M, Braithwaite S, Radcliff G. The development of a self-report scale. Health Visit. 1996.

[42] Høivik MS, Burkeland NA, Linaker OM, Berg-Nielsen TS (2013). The mother and baby interaction scale: a valid broadband instrument for efficient screening of postpartum interaction? A preliminary validation in a Norwegian community sample. Scand J Caring Sci.

[43] Sedgwick P (2012). Restricted randomisation. BMJ..

[44] Hawthorne J, Nicolau S (2017). Newborn Behavioural observations system: benefits and opportunities for integration into practice. J Heal Visit.

[45] McManus BM, Nugent JK (2011). Feasibility study of early intervention provider confidence following a neurobehavioural intervention for high-risk newborns. J Reprod Infant Psychol.

[46] Nygren P, Green B, Winters K, Rockhill A (2018). What’s happening during home visits? Exploring the relationship of home visiting content and dosage to parenting outcomes. Matern Child Health J.

[47] Kearney MH, York R, Deatrick JA (2000). Effects of home visits to vulnerable young families. J Nurs Scholarsh an Off Publ Sigma Theta Tau Int Honor Soc Nurs.

[48] Kristensen IH, Kronborg H (2018). What are the effects of supporting early parenting by enhancing parents’ understanding of the infant? Study protocol for a cluster-randomized community-based trial of the newborn behavioral observation (NBO) method. BMC Public Health.

