# Supplementary material for: What are the effects of supporting early parenting by newborn behavioral observations (NBO)? A cluster randomised trial
Source: BMC Psychol. 2020 Oct 16;8:107. doi: 10.1186/s40359-020-00467-5 (PMC7574292; doi:10.1186/s40359-020-00467-5)
Supplement: Supplementary file 1 — Additional file 1. [file 40359_2020_467_MOESM1_ESM.docx]

**Additional Table 1** Comparison of selected baseline information among participants and dropouts in intervention and comparison districts, respectively.

|  | Intervention districts | | |  | Comparison distrcts | | |  |
| --- | --- | --- | --- | --- | --- | --- | --- | --- |
|  | Participants | Dropouts | P |  | Participants | Dropouts | P | Interaction P* |
| *Follow-up, 3 months* |  |  |  |  |  |  |  |  |
| Maternal age (mean) | 30.5 | 29.9 | 0.07 |  | 30.2 | 30.1 | 0.82 | 0.10 |
| Short education (%) | 38,3% | 54.4% | <0.001 |  | 35.5% | 40.0% | 0.19 | 0.03 |
| Living alone (%) | 3.2% | 6.0% | 0.04 |  | 3.3% | 5.8% | 0.09 | 0.49 |
| First child (%) | 46.0% | 41.2% | 0.18 |  | 45.9% | 51.1% | 0.18 | 0.06 |
| KPCS (mean) | 41.2 | 41.5 | 0.23 |  | 41.4 | 41.4 | 0.97 | 0.22 |
| *Follow-up, 9 months* |  |  |  |  |  |  |  |  |
| Maternal age (mean) | 30,2 | 30.0 | 0.44 |  | 30.5 | 30.2 | 0.35 | 0.97 |
| Short education (%) | 38.1% | 54.4% | <0.001 |  | 38.8% | 45.6% | 0.02 | 0.76 |
| Living alone (%) | 4.0% | 3.8% | 0.87 |  | 3.5% | 3.4% | 0.57 | 0.53 |
| First child (%) | 46.6% | 48.0% | 0.68 |  | 47.4% | 41.7% | 0.06 | 0.11 |
| KPCS (mean) | 41.2 | 41.6 | 0.09 |  | 41.2 | 41.3 | 0.49 | 0.46 |
| * Interaction between intervention and participation, using linear regression for continuous baseline factors and logistic regression for binary factors. | | | | | | | | |
